# Nonstructural Proteins of Alphavirus—Potential Targets for Drug Development

**DOI:** 10.3390/v10020071

**Published:** 2018-02-09

**Authors:** Farhana Abu Bakar, Lisa F. P. Ng

**Affiliations:** 1 Singapore Immunology Network, Agency for Science, Technology and Research (A*STAR), Singapore 138648, Singapore; farhana_abubakar@immunol.a-star.edu.sg; 2 School of Biological Sciences, Nanyang Technological University, Singapore 636921, Singapore; 3 Department of Biochemistry, Yong Loo Lin School of Medicine, National University of Singapore, Singapore 117596, Singapore; 4 Institute of Infection and Global Health, University of Liverpool, Liverpool L69 7BE, UK

**Keywords:** alphavirus replication, nonstructural protein, protease inhibitors

## Abstract

Alphaviruses are enveloped, positive single-stranded RNA viruses, typically transmitted by arthropods. They often cause arthralgia or encephalitic diseases in infected humans and there is currently no targeted antiviral treatment available. The re-emergence of alphaviruses in Asia, Europe, and the Americas over the last decade, including chikungunya and o’nyong’nyong viruses, have intensified the search for selective inhibitors. In this review, we highlight key molecular determinants within the alphavirus replication complex that have been identified as viral targets, focusing on their structure and functionality in viral dissemination. We also summarize recent structural data of these viral targets and discuss how these could serve as templates to facilitate structure-based drug design and development of small molecule inhibitors.

## 1. Introduction 

Alphaviruses belong to the *Togaviridae* family [1]. They are arboviruses that are transmitted to humans through the mosquito species *Aedes aegypti* and *Aedes albopictus*, where they cause various diseases and can be broadly divided into arthritogenic [2] and encephalitogenic [3]. Old World alphaviruses such as chikungunya virus (CHIKV), o’nyong’nyong virus (ONNV), and sindbis virus (SINV) are arthritogenic and commonly cause febrile illness accompanied by rash, polyarthralgia and chronic arthritis [4,5,6,7,8]. Infection with New World alphaviruses such as Eastern equine encephalitis (EEEV) and Venezuelan equine encephalitis virus (VEEV), which are less prevalent in humans, are mostly associated with neurological disease [9,10]. In 2005, the re-emergence of CHIKV in several Indian Ocean islands led to massive outbreaks, affecting one-third of the inhabitants in La-Réunion alone [11]. To date, millions of people have been infected in more than 40 countries in Asia, Europe and the Americas [12]. Despite the medical threat posed by CHIKV, there is currently no approved antiviral treatment or vaccine for CHIKV infection, as well as for the other less prevalent alphaviruses. Treatments are usually symptomatic, with administration of non-steroidal anti-inflammatory drugs and analgesic agents to control fever and severe joint pains.

### Molecular Virology and Genome Organization

**Figure 1:**
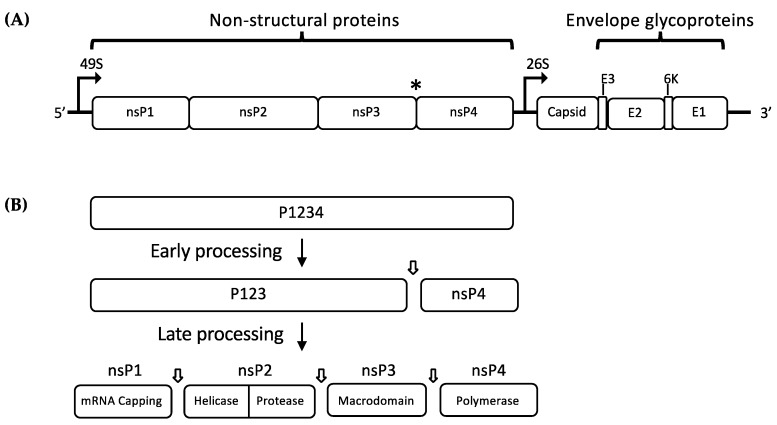
(**A**) Schematic representation of the alphavirus genome showing the RNA sequence open reading frames (ORFs). The (*) indicates the position of opal termination codon; (**B**) schematic representation of non-structural polyprotein (nsP2) processing by nsP2 protease. Early processing of P1234 produces P123 and nsP4 which associate to form the early replication complex (RC), which performs negative-sense RNA synthesis. P123 is further processed to produce the individual nsPs, which associate to form mature RC that regulates positive-sense RNA synthesis and transcription of subgenomic 26S RNA.

Alphaviruses are small, enveloped RNA viruses with a single-stranded, positive-sense RNA genome. The alphavirus genome is approximately 12 kb long and it consists of two open reading frames (ORFs): a 7 kb frame encoding the non-structural polyprotein and a 4 kb frame encoding the structural polyprotein (Figure 1A) [1,13]. The non-structural polyprotein is cleaved into four different proteins (nsP1, nsP2, nsP3, and nsP4) which are necessary for the transcription and translation of viral mRNA inside the cytoplasm of host cells. Figure 1B describes the stages of nonstructural polyprotein processing. Translation of the viral RNA by host cell translational machinery produces two non-structural protein (nsP) precursors (P123 or P1234). P1234 is expressed as a read-through of an opal termination codon at the end of nsP3 (Figure 1A) [14]. These precursor polyproteins are cleaved by a carboxyl-terminal protease domain of nsP2 [15]. Following translation of P1234, cleavage at the P3/4 junction occurs either in *cis* or *trans*, followed by the P1/2 junction which occurs in *cis* only [16]. Both P123+nsP4 and nsP1+P23+nsP4, together with some cellular proteins, form the early replication complex (RC), which preferentially synthesize negative strand viral RNA [17,18]. The final cleavage event at the P2/3 junction produces fully mature nsPs, which along with host cell proteins, forms the positive strand RC, switching RNA template for synthesis of positive-sense genomic (49S) and subgenomic (26S) RNAs. However, the correlation between P23 cleavage and the switch from negative- to positive-sense RNA production remains poorly understood.

The translation of 26S subgenomic positive sense RNA generates a single structural polyprotein, which is cleaved into five structural proteins: the Capsid (C), two major envelope glycoproteins E1 and E2, and two small cleavage products (E3, 6K) (Figure 1A). While the C protein is being autocatalytically cleaved off to encapsidate new positive sense RNA molecules, the envelope polyprotein precursor E3-E2-6k-E1 is translocated to the endoplasmic reticulum (ER). Host signalases process the polyprotein at the N- and C-terminal end of the 6k peptide, resulting in E3E2, 6k, and E1, where all anchored to the ER membrane. During export to the plasma membrane, the E3E2 precursor is cleaved by furin-like protease activity in the trans-Golgi system into E2 and E3 [19]. The nucleocapsid forms with the assembly of 120 dimers of the C protein, which buds at the cell membrane as spherical particles, acquiring a lipid envelope with embedded E1 and E2 glycoproteins [20,21]. Viral particles exhibit 80 trimeric spikes composed of heterodimers of E1 and E2, with E2 glycoprotein facilitating binding of the virus to cell surface receptors [22,23,24,25]. Upon receptor binding, the virus particle enters the host cell via clathrin-dependent endocytosis [26]. The acidic environment of the endosome results in the E1-mediated fusion of the viral envelope and endosomal membrane, followed by the release of nucleocapsid and uncoating of the viral genome in the cytosol [27,28].

In this review, the organization of alphavirus RC focusing on the function and structure of nsP2 protease will be illustrated. These proteins play important roles in the various replication stages of the viral genome. Notably, the conserved architecture of the nsP2 protease across the New and Old World alphaviruses, as determined by the recently solved structures of VEEV, SINV and CHIKV nsP2 proteases, also makes it an ideal target for designing specific and pan-alphavirus protease inhibitors [29,30,31,32].

## 2. Roles and Function of Non-Structural Proteins

### 2.1. Non-Structural Proteins (nsPs)

Key advances have been made to understand the biological aspects and pathogenesis of alphaviruses, using mainly Semliki Forest virus (SFV) and SINV as prototypes [33,34]. Many of the functions of the nsPs have been characterized on the basis of sequence comparisons and biochemical assays (Table 1), and evidence suggests their intrinsic interactions are essential for the formation of functional RCs [18,35,36,37].

#### 2.1.1. Non-Structural Protein 1 (nsP1)

The nsP1 protein is an mRNA capping enzyme that possesses both guanine-7-methyltransferase (MTase) and guanylyltransferase (GTase) activities, where they direct the methylation and capping of newly synthesized viral genomic and subgenomic RNAs [38,39,40,41]. The MTase motif in the N-terminal domain of nsP1 catalyzes the transfer of the methyl group from S-adenosylmethionine (AdoMet) to the N7 position of a GTP molecule (m^7^Gppp). GTase then binds the m^7^Gppp, forming a covalent link with a catalytic histidine (m7Gp–GTase) and releasing PPi. The GTase then transfers the m^7^Gp molecule to the 5′-diphosphate RNA to create m^7^GpppNp-RNA [42]. The resulting cap structure is essential for viral mRNA translation and prevents the mRNA from being degraded by cellular 5′ exonucleases. Following the N-terminal domain are features that allow the association of the nsP1 protein to cellular membranes. The presence of α-helical amphipathic loop and palmitoylation sites allow the nsP1 protein and nsP1-containing RC to anchor onto the plasma membrane, possibly through nsP1 interaction with the membrane’s anionic phospholipids [43,44,45,46,47].

#### 2.1.2. Non-Structural Protein 2 (nsP2)

**Figure 2:**
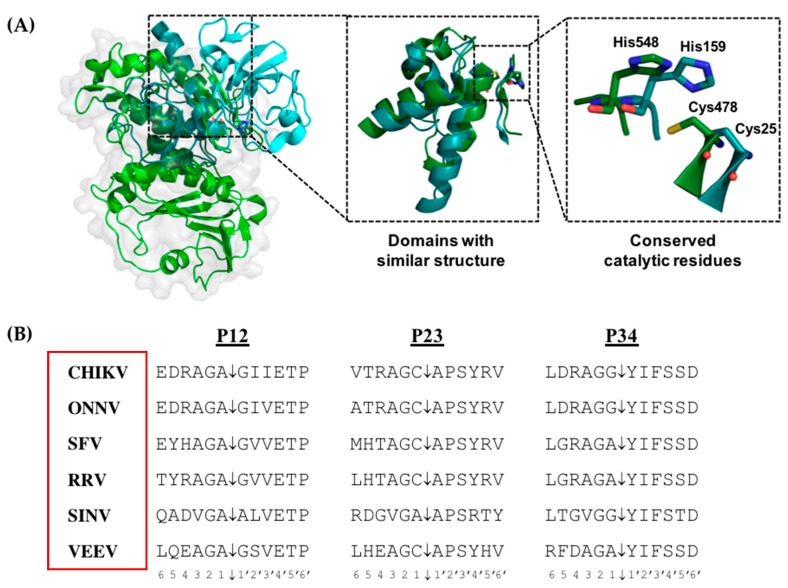
(**A**) Illustration of superposed structures of papain and chikungunya virus (CHIKV) nsP2 proteases (Protein Data Bank (PDB) 9PAD and 3TRK respectively). The structure of papain protease is presented as a solid, blue ribbon. The structure of CHIKV nsP2 protease is presented as solid, green ribbon. The domain common for both proteases is highlighted in dark colors and is enlarged subsequently to show the conserved catalytic dyad; (**B**) alignment of nsP2 cleavage sites. The nomenclature of Berger and Schechter is used to identify residues on the amino (P1, P2, etc.) or carboxy (P1′, P2′, etc.) termini of the scissile bond. The arrow indicates the location of the cleavage site. Cleavage sites between the non-structural proteins contain a common motif, AG(A/C)↓(G/Y/A) [55]. Abbreviations: Chikungunya virus (CHIKV), o’nyong’nyong virus (ONNV), semliki forest virus (SFV), ross river virus (RRV), sindbis virus (SINV) and Venezuelan equine encephalitis virus (VEEV).

The nsP2 protein possesses numerous enzymatic activities and functional roles. The N-terminal region contains a helicase domain that has seven signature motif of Superfamily 1 (SF1) helicases [48]. It functions as an RNA triphosphatase that performs the first of the viral RNA capping reactions [49,50]. It also functions as a nucleotide triphosphatase (NTPase), fueling the RNA helicase activity [50,51]. The C-terminal region of nsP2 contains a papain-like cysteine protease, which is responsible for processing the viral non-structural polyprotein (Figure 2A) [52,53,54]. The protease recognizes conserved motifs within the polyprotein (Figure 2B) [55]. This proteolytic function is highly regulated and is modulated by other domains of nsP2 [16]. The crystal structure has revealed two distinct domains. The N-terminal subdomain has a α/β-fold that is novel, unlike the structure of other known cysteine proteases. The C-terminal subdomain is an *S*-adenosyl-l-methionine-dependent (SAM) RNA methyltrasferase domain with a classical MTase fold, but enzymatically non-functional [29,30]. Recent crystal structure of VEEV’s enzyme surface has revealed that the predicted active site is in a major surface groove, which is likely to accommodate the substrate polyprotein to be cleaved [29,30]. The major enzyme groove may act as an enzyme mouth holding the protein to be processed. The alphavirus nsP2 protein has also been described as a virulence factor responsible for the transcriptional and translational shutoff in infected host cells and the inhibition of interferon (IFN) mediated antiviral responses contributing to the controlling of translational machinery by viral factors [56,57,58,59].

#### 2.1.3. Non-Structural Protein 3 (nsP3)

The precise role(s) of alphavirus nsP3 protein in the RC is less clear. The nsP3 protein has three recognized domains: the N-terminal macrodomain with phosphatase activity and nucleic acid binding ability, the alphavirus unique domain (AUD) and the C-terminal hypervariable domain [60]. The crystal structures of the macrodomain from CHIKV and VEEV were found to closely resemble the homologous *Escherichia coli* domain [60]. To date, the most well-defined structural information available are the protease region of nsP2 and the folded N-terminal region of nsP3 (Table 1). It has been solved to 2.85 Å resolution and has a zinc coordination site within the AUD [32]. The hypervariable domain has sequence features of natively unfolded proteins. It has been demonstrated that the deletion of this domain in SFV nsP3 resulted in low viral pathogenicity, suggesting its importance in viral RNA transcription regulation [61].

#### 2.1.4. Non-Structural Protein 4 (nsP4)

The nsP4 polymerase is the most highly conserved protein in alphaviruses, with the most divergent being >50% identical in amino acid sequence when compared with other alphaviral nsP4s [62,63]. The nsP4 contains the core RNA-dependent RNA polymerase (RdRp) domain at the C-terminal end, determined to be solely responsible for the RNA synthetic properties of the viral RC [64]. The RdRp participates in replicating the genomic RNA via a negative strand RNA and transcribing the 26S subgenomic RNA. The N-terminal domain is alphavirus-specific and may be partially disordered structurally. It appears to be important for the interaction with polyprotein P123 to form RCs that are capable of synthesizing minus strands from plus-strand templates [64,65,66]. Deletion and mutation studies of the RdRp domain of SINV nsP4 demonstrated terminal adenylyltransferase (TATase) activity, suggesting it has a potential role in maintenance of the 3′ poly-A tail at the 3′-end of positive-sense RNAs [66]. Comparison of the secondary structure of CHIKV RdRp with the structures of picornaviral polymerases showed a classical, basic RdRp architecture with well-defined fingers, palm containing the GDD active site and thumb domains [67]. A comprehensive review of the nsP4 was recently published that illustrated the fundamental functions of nsP4. It detailed the importance of P123 interaction with nsP4, the importance of RCs’ association with the cellular membrane and the possible interactions of RCs with host factors during viral replication [68].

### 2.2. Viral Target Proteins for Drug Development

Alphavirus replication is a delicate process, requiring specific protein-protein interactions among the nsPs and host factors to effectively form the highly organized RCs at early and late stages of infection. During infection, the formation of cytoplasmic vacuoles is induced in host cells. These vacuoles contain small cell membrane invaginations called spherules where the RC proteins nsP1 to nsP4, host factors as well as newly synthesized viral RNA localize [69,70,71,72,73]. These structures serve as compartments to facilitate virus propagation, by allowing spatial separation and regulation of RNA translation, replication and packaging of the viral genome. They protect viral RCs and genomic RNA from degradation by cellular proteases and prevent recognition by antiviral double-stranded RNA sensors, such as RIG-I and MDA-5 [74,75,76,77]. Although no 3-D reconstruction of alphavirus replication compartments has been published yet, there are multiple reports revealing the morphologies through electron tomography of other positive sense RNA viruses known to form similar replication sites [78,79,80,81]. Alphavirus RCs are widely accepted to reside on the invaginated cell membrane, with RNA replication taking place in the spherule lumen [70,82,83]. In recent years, attempts have been made to shed light into the late RC’s organization, with a number of studies adopting systems such as yeast two-hybrid [84,85], immunoprecipitation [86,87,88,89] and ELISA [85] to map the interactions among the nsPs. Six novel interactions are identified in CHIKV (nsP1-nsP1, nsP1-nsP2, nsP1-nsP3, nsP1-nsP4, nsP2-nsP4 and nsP4-nsP4) [84], some of which are similarly shown in SINV [88,89,90] and SFV [86,87]. It has also been demonstrated that nsP1 is involved in the recruitment of other nsPs into the spherules [87] and it’s membrane association is crucial for SFV replication [46]. These data suggest that nsP1′s interaction with all other nsPs is absolutely essential to keep the RC intact and functional. Therefore, nsP1 is an attractive target for drug development. Firstly, perturbing its affinity for the cell membrane could potentially inhibit it from anchoring in the spherules. This will eventually stop the recruitment of the other nsPs, thus preventing the initiation of RC formation. Secondly, disrupting the intrinsic interactions among the nsPs could prevent proper conformational arrangements of the RCs and this could ultimately impede viral replication. However, there is an information deficit in the understanding of nsP interactions and without this knowledge, targeted inhibition on RCs remains difficult. Future work will still need to address the relative importance of the arrangements between the nsPs in the RCs.

nsP2 protein is another excellent viral target for inhibition due to its role in viral replication and host evasion strategies [91]. nsP2 is multifunctional: it has RNA helicase, RNA triphosphatase, nucleoside triphosphatase and auto-protease activities. It is often described as an important co-factor for the maturation of viral RC [16,37,92]. The nsP2 protease function is especially of interest, as proteases of other viruses (such as Human Immunodeficiency Virus (HIV) and Hepatitis C Virus (HCV)) have been successfully targeted, leading to the development of FDA-approved inhibitors [93,94,95]. Although the sequence identity of the different alphavirus nsP2 proteases is very low, their active site residues are conserved [29]. Cys478 and His548 of CHIKV nsP2 protease (referred as Cys1013 and His1083 in [31]) are two residues that form the catalytic dyad (Figure 2A), where substrates with defined recognition sequences are cleaved (Figure 2B) [1,31,96,97,98]. It has been demonstrated that a Cys478 to Ala mutation produces inactive protease and completely abolishes CHIKV replication [98]. Recently, the first structure of a peptide-like E64d inhibitor-bound VEEV nsP2 protease (PDB entry 5EZS) was reported (Table 2) [30]. Although E64d is not a viable therapeutic candidate as it could only inhibit protease function and was ineffective in inhibiting viral replication, the structure provided invaluable insights into the roles of the catalytic residues and possible orientation of the substrates during catalysis. The inhibitor was shown to bind beneath a β-hairpin in the interface of the protease and SAM MTase domains. This further demonstrated that the SAM MTase domain is required for proteolysis, with at least three of its residues (Arg662, Lys705 and Lys706) being used by the cysteine protease for substrate binding and recognition. His546 (referred as His548 in CHIKV nsP2) in the protease domain is the only residue adopting a different conformation, with minimal overall structural changes observed when compared to the free enzyme. It was also suggested that the interaction of the carbonyl oxygen of the ester on E64d with the NH of Cys477 (referred as His478 in CHIKV nsP2) stabilized the transition state of the proteolytic reaction. These observations are crucial information as they emphasize the importance of residues within and around the active site cleft. Therefore, targeting these residues would be an applicable strategy to inhibit the enzyme function, which could consequentially inhibit viral replication. Moreover, nsP2 is also identified as a virulence factor. nsP2 plays a role in shutting off host cell mRNA transcription and/or translation [56,59]. A portion of the nsP2 is localized to the nucleus [99,100] and it inhibits host antiviral response by suppressing type I/II interferon-stimulated JAK/STAT signaling [101,102]. A recent study showed that mutations in the nuclear localization signal (NLS) of SFV nsP2 (^649^DDR^651^ and ^649^RDD^651^) completely blocked nsP2 from entering the nucleus and reduced SFV-induced cell death [103]. This was likely due to retention of nsP2 in the cytoplasm that prevented its association with host factors in the nucleus to shut off host antiviral functions. The NLS of nsP2 could be a good inhibitory target for preventing nsP2 translocation into the nucleus. However, this will only be effective against specific alphaviruses that use nsP2 to inhibit cellular mRNA transcription as it has been demonstrated otherwise for VEEV nsP2 [59].

## 3. Chemotherapeutics Targeting Viral Non-Structural Proteins

### 3.1. Antivirals against nsP2 Protease

The reemergence of CHIKV and its subsequent epidemics exemplify the threat alphaviruses currently have on human health. A comprehensive review of the antiviral efforts towards alphaviruses was recently published, where the various types of inhibitors discovered and developed as potential therapeutics were described [104]. It is of prime importance that resolved structures are employed in facilitating the discovery of novel drug compounds. The nsP2 protease’s function as a nonstructural polyprotein processor is absolutely essential for virus replication. Thus, inhibiting this function could potentially mitigate CHIKV infection. This strategy has successfully been used against several viruses like HCV and HIV and has led to the development of remarkably potent drugs [95,105,106,107]. With the recent availability of various alphavirus nsP2 protease crystal structures, they have become the most targeted viral domain for in silico drug design to date. Furthermore, crystal structures of the VEEV, CHIKV and SINV nsP2 protease domain Protein Data Bank (PDB) entries 2HWK [29], 5EZQ [30], 3TRK [31] and 4GUA [32] respectively] displayed highly conserved folds despite having low sequence identity, rendering it a potential target both for specific and pan-alphavirus protease inhibition. Bassetto et al. have effectively employed homology modeling and computer-aided drug design strategies to discover the first few potential compounds against CHIKV nsP2 protease, with the support of a combination of cell-based virus inhibition and cell-free protease assays [108]. Through the virtual screening of about five million commercially available compounds on the VEEV nsP2 protease structure, several were shown to selectively inhibit CHIKV-induced cell death. Compound 25, in particular, displayed the best antiviral activity (EC_50_ = 3.2 μM) and is predicted to target the central portion of the nsP2 protease active site (Table 2) [108]. Continuing from one of the other lead compounds by Bassetto et al., Das et al. described a set of related compounds, with a few capable of inhibiting CHIKV nsP2 protease as well as virus replication [109]. These results further prove that these compounds derived from in silico drug design are indeed nsP2 protease inhibitors. Many more potential inhibitors originating from the structure-activity relationship and molecular dynamics simulation strategies have been reported and illustrated to dock in the protease’s active site with good affinity [110,111,112]. However, the biological validation of their antiviral activity and/or target specificity is lacking. Aside from screening commercially available compound libraries, repurposing of FDA-approved protease inhibitors is another strategy that could accelerate the identification and development of specific and potent CHIKV protease inhibitors. Lopinavir and Nelfinavir, both potent HIV protease inhibitors, were validated to have modest antiviral activity on CHIKV (Table 2) (EC_50_ = 32 μM and 14 μM respectively). However, they are clearly toxic on the host cell as they display poor selectivity index values (CC_50_ = 44 μM and 22 μM respectively) [113]. Nonetheless, these inhibitors could serve as a stepping stone for the development of novel alphavirus protease inhibitors.

### 3.2. Inhibitors of Other nsPs

Inhibitors targeting other nsPs have also been reported, where their mechanisms of action on alphavirus replication were evaluated through reverse genetic mutation of drug-resistant mutants. A number of highly selective CHIKV and VEEV nsP1 capping enzyme inhibitors postulated to disrupt the GTase activity of nsP1 were reported recently [114,115,116]. MADTP-372 was demonstrated to be potent and selective in inhibiting the induction of cytopathic effect by CHIKV and VEEV [116]. Due to the close proximity of Pro34 residue (mutated in MADTP-372-resistant variant) to His37 residue (a putative acceptor for the guanylylation), it was postulated that MADTP-372 inhibited the GTase reaction by either blocking the binding of m^7^Gppp to nsP1 or the guanylylation process itself. However, due to the absence of structural information of alphavirus nsP1, other possible scenarios that involve indirect effects cannot be ruled out. Nucleoside analogs have also been proven to be effective against several alphaviruses. Notably, Ribavirin (a guanosine analog widely used against a number of other RNA viruses) demonstrated effective inhibition of CHIKV and SFV genome replication by depleting GTP pools [117,118,119]. The resulting GTP deficiency in host cells could prevent proper capping of newly synthesized viral mRNA by nsP1, and consequently allow cellular 5′ exonucleases to degrade the uncapped mRNA. The absence of the cap structure could also impede viral mRNA translation. In addition, Ribavirin has been proposed to directly inhibit nsP4 RdRp through its interaction with Cys483 residue, resulting in an increase in replication fidelity [120,121]. Another modified nucleoside analog, β-d-*N*^4^-hydroxycytidine (NHC), was effective in reducing the rates of VEEV release and its infectivity [122]. NHC is by far the most potent nsP4 inhibitor ever reported (EC_50_ = 0.426 µM, CC_50_ > 200 µM) and could potentially be a substitute for Ribavirin as it is inefficient in developing NHC resistant mutants. Another well-studied nsP4 inhibitor, Favipiravir (T-705; a purine analog approved in Japan for the treatment of influenza infections), was shown to exert broad-spectrum anti-alphavirus activity in vitro and provide protection in a mouse model of lethal CHIKV infection [123]. Favipiravir selectively inhibits CHIKV nsP4 RdRp function through its interaction with Lys291 residue. Interestingly, this particular lysine residue is conserved in the polymerases of other positive-sense RNA viruses. Thus this may provide an explanation for Favipiravir’s broad-spectrum antiviral activity. Through the screening of chemical compound libraries, Compound-A was also recently found to inhibit CHIKV infection. Compound-A was demonstrated as a specific nsP4 RdRp inhibitor that could potentially inhibit RdRp’s ribonucleotide selection function by targeting Met2295 residue [124]. It demonstrated potent CHIKV antiviral activity but exhibited very poor selectivity (EC_50_ = 1.29 µM, CC_50_ = 5.84 µM). Nonetheless, Compound-A could serve as a starting point for chemical modifications so as to reduce its toxicity.

## 4. Concluding Remarks and Perspectives

The current understanding of interactions through structural evaluation of the nsP2 protease domain has opened avenues for the development of specific inhibitors. It is by far the most well-defined out of the other nsP domains and its structural information has been inspirational for computational biologists and chemists alike in designing many series of compounds, mainly targeting its catalytic binding pocket. The representation of E64d inhibitor bound to the nsP2 catalytic pocket is a major advancement towards identifying the key residues that may be important for substrate binding and recognition [30]. However, the lack of structural information on the other viral nsPs makes understanding of the mode of action of inhibitors and rational designing of specific and efficient inhibitors challenging. It is worthwhile to note that for all nsP1 and nsP4 inhibitors discussed earlier, the direct interactions with their targeted residues were never demonstrated. In particular, obtaining the crystal structure of alphavirus nsP4 RdRp would allow more robust comparison of this polymerase to other viral RdRps and new relationships between RdRp-encoding viruses could be formed. Resolving the structure of the RC will also allow for a better understanding of the function of the polymerase and the entire RC.

## Figures and Tables

**Table 1:** Non-structural protein (nsP) domain functions and available crystal structures.

Non-Structural Protein	Domain Function	Virus	PDB ID	Reference
nsP1	mRNA capping	SFV	1FW5	[45]
nsP2	NTPase/HelicaseProtease	- VEEV CHIKV SINV	- 2HWK, 5EZQ 3TRK 4GUA	- [29,30] [31] [32]
nsP3	Macrodomain	VEEV CHIKV SINV	3GQE 3GPG 4GUA	[60] [60] [32]
nsP4	RNA-dependent RNA Polymerase	-	-	[32]

PDB: Protein data bank; SFV: Semliki Forest virus; VEEV: Venezuelan equine encephalitis virus; CHIKV: chikungunya virus ; SINV: sindbis virus.

**Table 2:** List of compounds shown to inhibit nsP2 protease activity.

Molecule	Structure	Virus	EC_50_	Reference
Compound 25	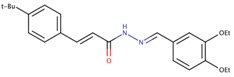	CHIKV	3.2 μM	[108]
Compound 1c	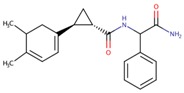	CHIKV	27 μM	[109]
Nelfinavir	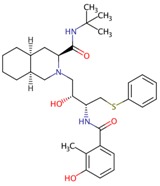	CHIKV	14 μM	[113]
E64d—cysteine protease inhibitor	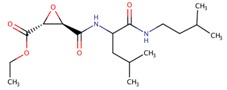	VEEV	ND	[30]

ND: not determined.
